# Test-Retest Reliability of fMRI Brain Activity during Memory Encoding

**DOI:** 10.3389/fpsyt.2013.00163

**Published:** 2013-12-09

**Authors:** David J. Brandt, Jens Sommer, Sören Krach, Johannes Bedenbender, Tilo Kircher, Frieder M. Paulus, Andreas Jansen

**Affiliations:** ^1^Section of Brainimaging, Department of Psychiatry and Psychotherapy, Philipps-University Marburg, Marburg, Germany

**Keywords:** memory encoding, fMRI, lateralization, laterality, hemispheric dominance, test-retest, reliability, ICC

## Abstract

The mechanisms underlying hemispheric specialization of memory are not completely understood. Functional magnetic resonance imaging (fMRI) can be used to develop and test models of hemispheric specialization. In particular for memory tasks however, the interpretation of fMRI results is often hampered by the low reliability of the data. In the present study we therefore analyzed the test-retest reliability of fMRI brain activation related to an implicit memory encoding task, with a particular focus on brain activity of the medial temporal lobe (MTL). Fifteen healthy subjects were scanned with fMRI on two sessions (average retest interval 35 days) using a commonly applied novelty encoding paradigm contrasting known and unknown stimuli. To assess brain lateralization, we used three different stimuli classes that differed in their verbalizability (words, scenes, fractals). Test-retest reliability of fMRI brain activation was assessed by an intraclass-correlation coefficient (ICC), describing the stability of inter-individual differences in the brain activation magnitude over time. We found as expected a left-lateralized brain activation network for the words paradigm, a bilateral network for the scenes paradigm, and predominantly right-hemispheric brain activation for the fractals paradigm. Although these networks were consistently activated in both sessions on the group level, across-subject reliabilities were only poor to fair (ICCs ≤ 0.45). Overall, the highest ICC values were obtained for the scenes paradigm, but only in strongly activated brain regions. In particular the reliability of brain activity of the MTL was poor for all paradigms. In conclusion, for novelty encoding paradigms the interpretation of fMRI results on a single subject level is hampered by its low reliability. More studies are needed to optimize the retest reliability of fMRI activation for memory tasks.

## Introduction

Hemispheric specialization is a basic principle of human brain organization. Although functional asymmetries of brain functions were already known since the middle of the 19th century, the underlying mechanisms are not completely understood (1). In particular, we do not have precise models that explain which factors are responsible for hemispheric specialization, why the degree of lateralization varies from individual to individual, and how the brain integrates processes that are lateralized to opposite hemispheres. The investigation of brain lateralization is not only important from a neuroscientific perspective, but has also clinical implications, for instance to better assess the long-term effects of a stroke or of a neurosurgical intervention. For instance the effects of damage to the left hemisphere on language performance might be less severe in individuals with bilateral or right-dominant language lateralization (2, 3). Also many psychiatric disorders, in particular schizophrenia, have been associated with altered brain lateralization (4). Any theory trying to describe the neural correlates of schizophrenia therefore has also to incorporate aspects of variability of hemispheric dominance.

The development of functional imaging techniques, in particular functional magnetic resonance imaging (fMRI), has made it possible to study non-invasively the neural correlates of cognitive processes. FMRI can now be used to develop and test models of hemispheric lateralization (5, 6). Comprehensive models of brain lateralization however do not only have to describe the hemispheric specialization of one specific brain function, but also have to account for the interaction of the hemispheric dominance of different brain functions such as language, spatial attention, and memory (7). This makes it necessary to robustly determine the hemispheric lateralization of these brain functions on the single subject level (7–9). The interpretation of fMRI results in individual subjects however is often hampered by the low test-retest reliability of the data (10). In contrast to the high relevance of the reproducibility of brain activity measures of memory processes, both for basic neuroscientific and clinical questions, surprisingly few previous fMRI studies had so far explicitly assessed the test-retest reliability of memory paradigms (10). These results have been mixed. While some studies reported relatively high test-retest reliability related to memory encoding (11, 12), others showed low reliability (13).

In the present study we therefore investigated the test-retest reliability of fMRI brain activation related to an implicit memory encoding task. As memory task, we chose a commonly applied novelty encoding paradigm contrasting stimuli that are either “new,” that is, shown only once during the experiment, or “old,” that is, shown several times (14, 15). Under the assumption that the encoding of known stimuli poses less demands on the neural network underlying memory functions, the comparison of both conditions enables to visualize brain regions that are involved in the encoding of information [“novelty encoding”; for a discussion of other memory paradigms see Ref. (16)]. The lateralization of the memory encoding network is determined among other things by the verbalizability of the memorized material (14). Encoding of verbal stimuli preferentially relies on left-hemispheric brain regions, encoding of visuospatial (non-verbal) material on right-hemispheric areas.

To be able to also assess brain lateralization, we used three different stimuli classes that differ in their verbalizability (word, scenes, fractals). According to the results of Golby and colleagues (14), we expected left-lateralized brain activity for words, bilateral activity for scenes, and right-lateralized activity for fractals. Test-retest reliability of fMRI brain activation was assessed by the intraclass-correlation coefficient (ICC), describing the stability of inter-individual differences in the brain activation magnitude over time. A specific focus of our analysis was the reliability of brain activation within the medial temporal lobe (MTL) since this regions is considered as most critical for declarative memory encoding (17–19).

## Materials and Methods

### Subjects

Twenty healthy subjects (13 men), aged 20–37 years (mean age = 25.6 ± 4.0 years), participated in the study. Written informed consent was obtained prior to participation according to the declaration of Helsinki. The study was approved by the ethics committee of the medical faculty of the University of Marburg. All participants were native German speakers, right-handed according to the Edinburgh handedness inventory (20) and had completed the equivalent of a high school degree (“Abitur”). None of the subjects had a history of neurological or psychiatric illnesses, brain pathology, or abnormal brain morphology on T1-weighted MR images. To investigate the test-retest reliability, subjects were scanned twice on two sessions separated by 35 days on average (range 20–57 days). Five participants were not available for a second measurement.

### Experimental procedure

The memory paradigm consisted of two conditions in which known and unknown stimuli were presented in alternating blocks. The known stimuli (henceforth referred to as “old”) had repeatedly been presented before the actual experiment, the unknown stimuli (henceforth referred to as “new”) were not shown before. Subjects were not informed about the existence of different conditions.

**Figure 1:**
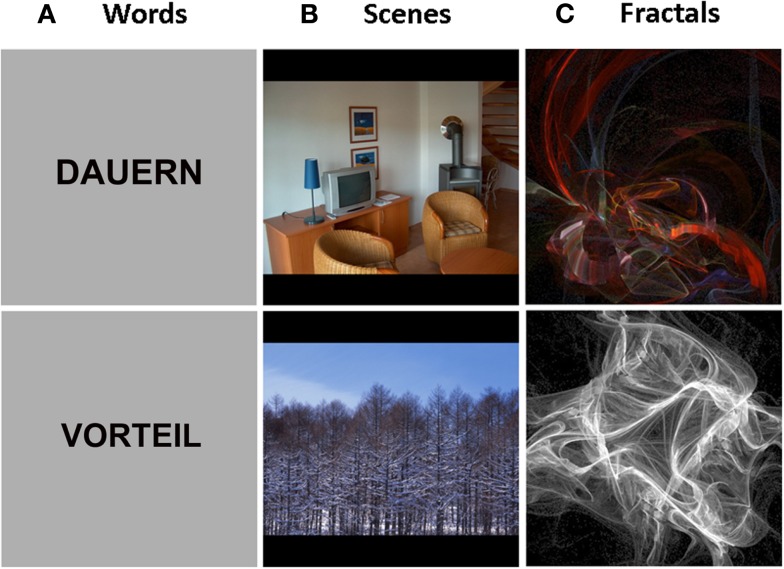
**Example-stimuli for each stimulus type**. Words **(A)**, indoor/outdoor scenes **(B)**, and fractals**(C)**.

We used three types of stimuli that varied in their verbalizability: words (high verbalizability), photographs of indoor and outdoor scenes (intermediate verbalizability), and fractals (low verbalizability). An example of each stimulus type is presented in Figure 1 (more information on the creation of the stimulus material is presented in Appendix). The different stimulus material was presented in separate sessions. The order of stimulus type (words/scenes/fractals) and novelty (old/new) was counterbalanced across subjects. For the second measurement, we used the same order as in the first measurement, but a set of different stimuli. All stimuli were presented visually using the software package *Presentation* (NeuroBehavioral Systems Inc.).

Each session was divided in two parts, a “familiarization” phase and a “measurement” phase. In the familiarization phase, subjects viewed 10 stimuli that were later used as old stimuli. Each old stimulus was presented 10 times. In the measurement phase, subjects were presented with 60 old stimuli (each old stimulus was thus shown six times) and 60 new stimuli (each new stimulus was shown only once) in alternating blocks of variable length. Stimuli were presented for 2 s, followed by a fixation cross shown for 2 s. The epoch length varied between two and six stimuli, with an average block length of five stimuli. To ensure a high level of attention, the subjects were given a material-specific task which consisted of verb/noun – decision in the verbal paradigm, indoor/outdoor – decision in the scenes paradigm and colored/non-colored-decision in the fractal paradigm. All decisions were given by pressing one of two buttons of a MR-compatible response box using their right hand. The subjects were not explicitly instructed to memorize the presented stimulus material.

### MRI data acquisition

MRI data was acquired on a 3-T Tim Trio MR scanner (Siemens Medical Systems, Erlangen) at the Philipps-University Marburg. Functional images were collected with a T2* weighted echo planar imaging (EPI) sequence sensitive to BOLD contrast (64 × 64 matrix, FOV 230 mm, in plane resolution 3.6 mm, 38 slices, slice thickness 3.6 mm, TR = 2.5 s, TE = 30 ms, flip angle 90°). Slices covered the whole-brain and were positioned transaxially parallel to the anterior–posterior commissural line (AC–PC). Two hundred and fifteen functional images were collected in the measurement phase of each session.

### MRI data analysis

*S*PM8 (http://www.fil.ion.ucl.ac.uk/spm) standard routines and templates were used for the fMRI data analysis. The functional images were realigned, normalized (resulting voxel size 2 mm × 2 mm × 2 mm), smoothed (applying a 8-mm full-width-at-half-maximum, FWHM, isotropic Gaussian filter), and high-pass filtered (cut-off period 128 s). Statistical analysis was performed in a two-level, mixed-effects procedure separately for each stimulus class and each measurement. At the subject level, the BOLD responses for the encoding of new and old stimuli, respectively, were modeled by the canonical hemodynamic response function of SPM8 and its time derivative. The six realignment parameters of head motion were included in the statistical model to account for residual head movement. Contrasted parameter estimate images (con-images), describing brain activation differences between new and old stimuli (“new > old”), were calculated for each subject.

#### Analysis 1: brain activation at the group level

In a first step, we analyzed for each paradigm brain activation at the group level. We calculated separately for each paradigm and each session one-sample *t*-tests, using the con-images obtained in the single subject analysis as input data. Anatomical localization of brain activity was assessed using both the WFU-PickAtlas (21) and the SPM Anatomy Toolbox (22). We expected for all three paradigms for the contrast “new > old” brain activity in the MTL. Analogous to the results of Golby and colleagues (23), activity of the MTL was supposed to be left-lateralized for the encoding of words, right-lateralized for the encoding of fractals, and bilateral for the scenes paradigm. Hemispheric lateralization of brain activity in the MTL was assessed by a lateralization index (LI). Several approaches have been established to calculate a LI [for a discussion, see Ref. (24)]. We calculated the LI by the formula

$$\text{LI}=\left(A_{\text{L}}-A_{\text{R}}\right)∕\left(A_{\text{L}}+A_{\text{R}}\right),$$

where *A*_L_ and *A*_R_ refer to measures of fMRI activity in the left (L) and right (R) MTL. The MTL was defined as the hippocampus, the parahippocampus and the amygdala, using the WFU-PickAtlas (dilation factor 2). LI values can range from −1 (absolutely right-lateralized brain activity) to +1 (absolutely left-lateralized brain activity). As measures of activity, we used the number of active voxels above a statistical threshold *p*. Since the number of active voxels is strongly depending on the chosen threshold, we calculated the LI for a range of statistical thresholds (*p* = 0.001, *p* = 0.01, *p* = 0.05). The reliability of the MTL activation was qualitatively assessed for each paradigm by the analysis of the overlap of activated brain regions.

#### Analysis 2: retest reliability of brain activation

In a second step, we assessed the main question of the present study, that is, the test-retest reliability of the individual activation strength of brain activity for each paradigm. As a measure of retest reliability, we applied the ICC. The ICC describes the stability of inter-individual differences in brain activation magnitude over time. Mathematically, this coefficient sets within-subject variance $\left(\sigma_{\mathrm{within}}^{2}\right)$ in relation to between-subject variance $\left(\sigma_{\mathrm{between}}^{2}\right).$ We used the ICC(3,1)-type (25) computed as

$$\mathrm{ICC}=\left(\sigma_{\mathrm{between}}^{2}-\sigma_{\mathrm{within}}^{2}\right)∕\left(\sigma_{\mathrm{between}}^{2}+\sigma_{\mathrm{within}}^{2}\right).$$

The variance components were calculated by the individual contrast values (i.e., con-images) separately for each session. ICC values range from −1 to 1. According to established conventions, reliability will be classified as “poor” for ICC ≤ 0.4, as “fair” for 0.4 < ICC ≤ 0.6, as “good” for 0.6 < ICC ≤ 0.8, and as “excellent” for ICC > 0.8 (26, 27).

Intraclass-correlation coefficients can be calculated both on a voxel-by-voxel basis (“Voxel-ICCs”) and on a regions of interest (ROI) basis (“ROI-ICCs”). In a first approach, we calculated ICCs for each voxel using the matlab-based ICC toolbox provided by Caceres and colleagues (26). The calculation of ICCs on a voxel basis is the most flexible approach, since it allows testing for retest reliabilities in the whole-brain, outside specific ROIs. It furthermore enables to relate the reliability of brain activity (expressed by the ICC) to the strength of brain activity (expressed by the *t*-value). For specific ROIs, the ICC can subsequently be expressed as the median value of the distribution of the ICC in the ROI.

As shown in the results section, the overall test-retest reliability of all paradigms was below 0.40 and therefore had to be classified as poor. One reason might be that ICC maps which are calculated voxel-by-voxel are relatively prone to random noise. In a second approach, we therefore also calculated ICCs directly for specific ROIs. In this approach, activation values from the individual con-images were first averaged within a ROI, before an ICC was calculated. Although this proceeding is less flexible than the calculation of ICC maps, it is supposed to decrease random noise due to the averaging of activation values. Analogous to the approach described by Caceres and colleagues (26), we applied two different methods to sample the voxels from which the contrast values were extracted: (i) the mean value of all voxels in a ROI, (ii) the median value of all voxels in a ROI. As ROIs we chose on the one hand the left and right MTL, the main regions of interest in the present study, on the other hand also a reference region since brain activity in the MTL, in particular in the anterior hippocampus, is known to be affected by susceptibility artifacts. As reference regions, we used the left inferior frontal gyrus (Brodmann areas 44/45) for the words paradigm, and the left and right fusiform gyrus, respectively, for the fractals and scenes paradigms since these regions were most strongly activated by the respective paradigms. All ROI masks were created from the brain activation pattern at the group level, in order to match the ROI most closely to the activation maxima.

## Results

### Group activation pattern

**Figure 2:**
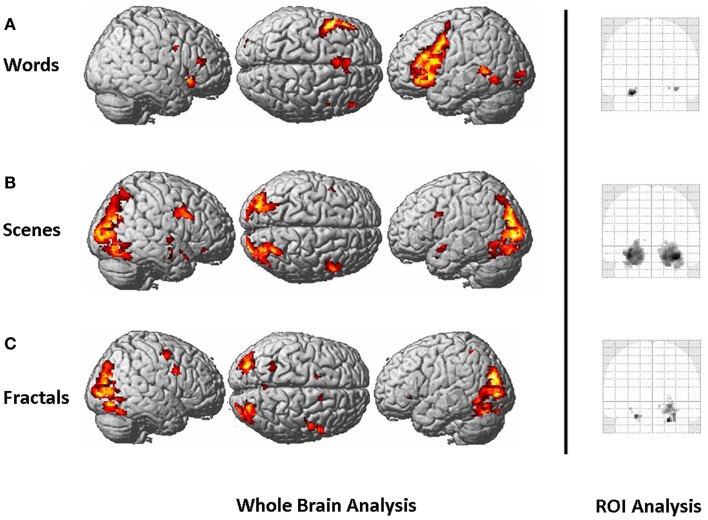
**Left: whole-brain activation pattern, as assessed by a mixed-effects group analysis (contrast: “new > old,” first session), for the words paradigm (A), the scenes paradigm (B), and the fractals paradigm (C)**. Right: ROI analysis of the MTL, defined as hippocampus, parahippocampus, and amygdala. Brain activation is presented as “glass brain projection” of the standard SPM8 MNI template. T-maps for the scenes and the fractals paradigms are thresholded at *p* < 0.001, uncorrected. For the whole-brain analysis, an (arbitrary) cluster size threshold of 20 contiguous voxels was applied. This threshold is not based on any procedures to correct for multiple testing, but rather serves for illustrational purposes. For the words paradigm, we present the results at a more liberal threshold (*p* < 0.01, uncorrected) since at *p* < 0.001 brain activation was only detected in the left prefrontal cortex.

The whole-brain activation pattern is presented for all paradigms in Figure 2. For the words paradigm, we found a left-lateralized brain activation network, with main activation centers located in the prefrontal cortex, the supplementary motor area, the inferior parietal cortex, the MTL, and the cerebellum. For the scenes paradigm, main activation clusters were found in the visual cortex, the bilateral MTL, and right prefrontal areas. The fractals paradigm activated a similar network, with the strongest activation centers in the visual cortex and the MTL.

For all three paradigms, we found brain activation of the MTL (Figure 2 right). Hemispheric lateralization was left-lateralized for the words paradigm, bilateral to right dominant for the scenes paradigm, and right-lateralized for the fractals paradigm. The LIs describing hemispheric lateralization of MTL brain activity are presented in Table 1.

**Table 1:** **Lateralization index’s calculated for different statistical thresholds *p* describing hemispheric lateralization of MTL brain activity for the words paradigm (A), the scenes paradigm (B), and the fractals paradigm (C)**.

Paradigm	*p*-Value	Active voxels (left MTL)	Active voxels (right MTL)	LI
Words	0.001	2	0	1.00
	0.01	73	10	0.76
	0.05	341	97	0.56
Scenes	0.001	1447	1676	−0.07
	0.01	2113	2309	−0.04
	0.05	2588	2640	−0.01
Fractals	0.001	26	96	−0.59
	0.01	269	717	−0.45
	0.05	936	1460	−0.22

A qualitative analysis of the overlap of brain activation at the group level shows that for the words and the scenes paradigm the same network was activated in both measurements (Appendix). For the fractals paradigm however, only weak brain activation was found during the second measurement, even at low significance thresholds (*p* < 0.01 uncorrected). One has to further lower the significance thresholds to see that, in principle, also during this paradigm the same network is activated in both sessions.

### Test-retest reliability

**Figure 3:**
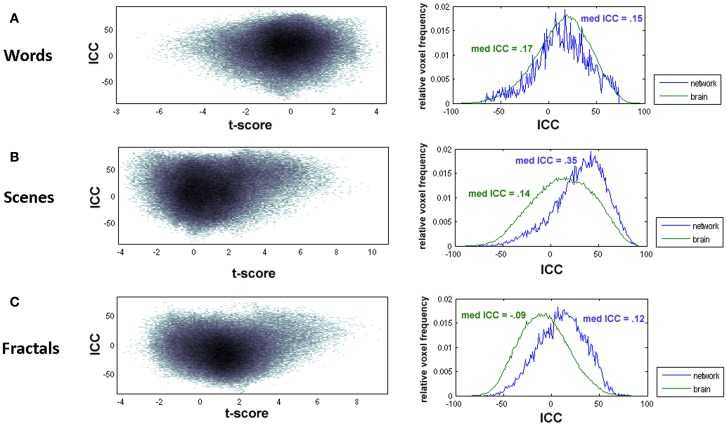
**Left: joint probability distribution of voxel-wise *t*-values and associated ICC values**. Right: ICC frequency distributions for the whole-brain (green) and for the voxels in the activated network (blue). The “activated network” was defined based on the results from the first measurement. Voxels were classified as active if they had *t*-values *t* > 3.79 (corresponding to *p* < 0.001) (scenes and fractals paradigms) and *t* > 2.60 (corresponding to *p* < 0.01) (words paradigm), respectively. Both diagrams are presented for the words paradigm **(A)**, the scenes paradigm **(B)**, and the fractals paradigm **(C)**.

In a first step, test-retest reliability was analyzed voxel-by-voxel. Whole-brain joint probability distributions showed an association between *t*-values and ICCs (Figure 3 left). ICCs were for all paradigms generally higher within brain areas showing strong activation (high *t*-values) or “deactivation” (high *t*-values for the opposite contrast, “old > new”). Thus, brain activity measures within encoding-relevant networks, that is, in brain areas significantly more or significantly less active in the “New”- than in the “Old“-condition, tended to be more reliable (Figure 3 right). As an exception, the ICC frequency distribution for the words paradigm had overall higher reliability values than the distribution for the whole-brain, showing that for this paradigm the overlap of voxels with high ICC values and the activated network is low. The overall reliability, expressed by the ICC distributions, was poor, in particular for the words and the fractals paradigm. For the fractals paradigm, the median ICC for the activated network was 0.12 (whole-brain: median ICC = −0.09), for the words paradigm the median ICC was 0.15 (whole-brain: med ICC = 0.17). The reliability values of the scenes paradigm were higher in comparison to the other paradigms. However, also for this paradigm median ICC values were below 0.4 (activated network: 0.35, whole-brain: 0.14) and thus had to be classified as poor.

In a second step, test-retest reliability was analyzed for specific ROIs. Activation values were first averaged within a ROI, then a ICC was calculated. As ROIs, we chose on the one hand the left and right MTL, on the other hand a reference region. As reference ROI, we chose that region that was on the group level most strongly activated. For the scenes and the fractals paradigm, we chose the left and right fusiform gyrus, respectively. For the words paradigm, only one reference ROI was chosen, the left prefrontal cortex, since no comparable activation was found in the right hemisphere. ICCs calculated on a ROI basis are presented for all paradigms in Table 2. Most ICCs were <0.4, independent of the specific calculation method, thus indicating poor reliability. Only for the scenes paradigm, the reliability for the left fusiform gyrus was slightly higher (0.42–0.45). The lowest reliability values were obtained for the fractals paradigm.

**Table 2:** **For each paradigm, ROI-based ICCs were calculated for four different ROIs: the left MTL, the right MTL, and two reference regions (Ref ROI)**.

Paradigm	Method	Left MTL	Right MTL	Ref ROI 1	Ref ROI 2
Words	Mean	0.01	0.30	0.16	–
	Median	−0.03	0.29	−0.08	–
Scenes	Mean	−0.07	0.03	0.42	0.28
	Median	0.19	0.03	0.45	0.28
Fractals	Mean	−0.53	−0.55	−0.13	−0.10
	Median	−0.53	−0.56	−0.11	−0.10

*As reference region, we chose the left (Ref ROI 1) and right fusiform gyrus (Ref ROI 2) for the scenes and fractals paradigm, and the left prefrontal cortex (Ref ROI 1) for the words paradigm. Activation values were calculated either by the mean or the median of the activation values of all voxels in the ROI*.

## Discussion

Functional magnetic resonance imaging has become an important tool in memory research. Functional imaging of memory processes is increasingly used to develop and test models of hemispheric lateralization (28), but is also applied in the clinical context, for instance in the pre-operative assessment of patients with MTL epilepsy (14). For both applications, reliability of brain activity is crucial. In the present study, we therefore determined the test-retest reliability of three commonly applied implicit memory paradigms that differed in the verbalizability of the encoded material (words, scenes, fractals). At the group level, both brain activation and hemispheric lateralization patterns were consistent with previous reports on memory encoding (14, 28). With regard to hemispheric dominance, the brain activation in the MTL was left-lateralized for the encoding of words, bilateral for the encoding of scenes, and right-lateralized for the encoding of fractals. We found reproducible brain activation in extended networks related to the encoding of the specific stimulus material at the group level. In contrast, a quantitative assessment of test-retest reliability on the single subject level using ICCs showed poor reliabilities for all paradigms, both for the overall activated brain network and for selected ROIs. Sole exception was the scene encoding task, for which reliability of brain activation might be considered as “fair,” at least in strongly activated reference regions. In the following, we will first discuss the methodology we have used to assess test-retest reliability, then evaluate our results in the context of the existing literature.

### Methodological considerations

Test-retest reliability of fMRI results can be investigated in a number of ways. The most commonly applied methods are the investigation of the overlap of activated voxels and the use of ICCs (10). The cluster overlap method analyses what set of voxels are activated during both test and retest sessions. Its main limitation is the dependency on the applied statistical thresholds used to define which voxels are “active,” limiting its overall practicability (24). In the present study, we nevertheless used this method as the most straightforward approach to qualitatively assess on the group level whether a specific task activates the same network in repeated measurements. At the group level, we found reproducible brain activation in extended networks related to the encoding of the specific stimulus material. For all paradigms, the same network was activated in both sessions.

The main goal of the present study was to investigate whether the strength of brain activation, in particular for the MTL, was a stable marker between test scan and retest scan on the individual subject level. The standard method to quantify this facet of reliability is the use of an ICC. The ICC assesses fMRI activation reliability by comparing the between-subject variance of the activation magnitude to the total variance. It is a more stringent criterion, especially when applied on a whole-brain, voxel-wise basis, than simple extent reliability since it is necessary to replicate the exact degree of activation (and not simple what survives thresholding).

For fMRI data, ICCs can be calculated in different ways. On the one hand, it is possible to calculate an ICC for every voxel, on the other hand one may use averaged activation values in specific ROIs to quantify reliability for selected brain regions. In a first step, we calculated ICCs for each voxel. The calculation of ICCs on a voxel basis allows to test for retest reliabilities outside specific ROIs and enables to relate the reliability of brain activity (expressed by the ICC) to the strength of brain activity (expressed by the *t*-value) (26, 27). Since within a predefined ROI several different brain activation clusters may exist (24, 29), the voxel-wise calculation of ICCs has the further advantage that it avoids the averaging of functionally distinct activations. For all three paradigms, the test-retest reliability using ICCs showed poor reliabilities, not only with regard to brain activity in the MTL, but also for both the overall activated brain network. For the fractals paradigm, the median ICC for the activated network was 0.12 (whole-brain: median ICC = −0.09), for the words paradigm the median ICC was 0.15 (whole-brain: median ICC = 0.17). For the fractals paradigm, the low overall reliability can be most likely explained by the weak activation strength of the second measurement compared to the first measurement (see Appendix). The reliability values of the scenes paradigm were higher in comparison to the other paradigms. However, also for this paradigm median ICC values were below 0.4 (activated network: 0.35, whole-brain: 0.14) and thus had to be classified as poor.

Although the voxel-wise calculation of ICCs is a flexible approach, it is not without problems, most of all due its susceptibility to the effects of random noise. Another approach to calculate ICCs is to first calculate activation values in predefined ROIs, than to calculate one ICC for each region from these averaged activation values. On the one hand, this approach is less prone to the effects of noise since it first averages across a larger set of voxels. On the other hand, it is also less dependent on the effects of small misregistrations during the normalization process. In a second step, we therefore also calculated ICCs in predefined ROIs for averaged activation values. As ROIs, we chose on the one hand the left and right MTL since this brain region was the main focus of the present study. Since it is known however that the assessment of brain activity in the MTL is often impeded by susceptibility artifacts that potentially may decrease the reliability of brain activation in this specific region, on the other hand we also assessed test-retest reliability in other reference regions. As reference region, we chose the maximally activated brain areas due to the correlation of activation strength and reliability. The most strongly activated brain regions were left inferior frontal gyrus for the words paradigm, and the left and right fusiform gyrus, respectively, for the other paradigms. However, also the second approach did not yield higher reliability values, with the exception of the left fusiform gyrus for the scenes paradigm (ICC = 0.42–0.45). The most likely explanation for the higher reliability values of the scenes paradigm in comparison to the other two paradigms is the overall higher activation for the contrast of interest (“new > old”). For the words paradigm, we had to lower the significance threshold to *p* < 0.01 (uncorrected for multiple comparisons) to find activation in other brain regions as the left inferior frontal gyrus. This might be explained by differences in encoding depth between the stimulus categories. “New” words are ecologically more familiar than for instance “new” scenes. For the fractals paradigm, brain activation during the second measurement was found only at extremely liberal thresholds (*p* < 0.05 uncorrected).

The ability of fMRI to detect meaningful and reproducible signals is limited by a number of factors that add error to each measurement, e.g., thermal noise, system noise in the scanner, physiological noise from a subject, subject motion, non-task related cognitive processes, changes in cognitive strategy over repeated measurements (30). It is therefore generally accepted that fMRI is a relatively noisy measurement method with a characteristically low signal-to-noise ratio (10), making it crucial that any fMRI study that investigates the reliability of brain activation has to ensure that all easily avoidable sources of error variance between sessions are excluded. We worked at a stable scanner environment, used imaging sequences that were known from previous experiments to be able to elicit robust brain activation of the MTL, and used relatively short retest intervals. One might have further standardized both measurement sessions, for instance by making sure that all subjects are measured at the same time of day or by making sure that subjects did not consume any alcohol or nicotine at least 1 day before measurement. However, in the overall view our measurements adhered to typical standards for fMRI studies, thus representing a realistic situation that will also be encountered in routine research and clinical settings.

### Evaluation of results

In the present study we assessed the test-retest reliability of fMRI brain activation related to implicit memory encoding, with a specific focus on brain activity in the MTL. Test-retest reliability of MTL brain activity was poor for all paradigms. In addition, two of three paradigms (words paradigm, fractals paradigm) yielded poor reproducibility of brain activation also for the overall activated network and even within the strongest activated reference regions. Therefore the low reproducibility of brain activation is not limited regions prone to susceptibility artifacts (such as the anterior hippocampus), but constitutes a general problem of the paradigms.

In contrast to the words and the fractals paradigm, the scenes paradigm had acceptable reproducibility characteristics, at least for the overall activated network and the reference regions. The reason that this paradigm performs better with regard to test-retest reproducibility is most likely caused by the stronger brain activation differences (as indicated by higher *t*-values) between the “new” and “old” condition. As shown in Figure 3, higher *t*-values are typically associated with higher ICC values. In contrast, the activation differences between “old” and “new” words are much smaller, most probably because words that are presented for the first time (that is, within the “new” condition) are already well known.

Although it is now widely accepted that fMRI provides valuable insights into the human brain, also on the individual subject level, there is no consensus yet on how reliable fMRI results are (10, 30). The analysis of the reliability of imaging data however is not only important for the pursuit of scientific truth, but perhaps even more for clinical applications. Although many clinical research groups have published fMRI studies that assessed hemispheric specific memory related brain activation in the MTL [e.g., Ref. (13–15, 23, 31, 32)], even supporting the application of memory tasks for clinical purposes (23), interestingly only few studies explicitly also assessed the test-retest reliability of these memory paradigms. In the functional imaging literature, there is an increasing interest to find non-invasive imaging biomarkers than can objectively evaluate for instance disease status or progression. Although fMRI is a promising tool, missing reliability is one major problem for the use as an individual test-retest biomarker (33). The insufficient reliability of fMRI paradigms might also help to explain difficulties in reproducing initially promising findings and contribute to non-findings in context of examining relatively small effect sizes in imaging genetics studies (34–36).

The results of previous reliability studies on memory encoding have been mixed. While some studies reported relatively high test-retest reliability related to memory encoding (11, 12), others showed limited reliability (13). Our results are at first glance in line with reports from Harrington and colleagues who also reported moderately high reproducibility values for a scene encoding task and low reliability for pattern encoding and word-pair encoding tasks. However, it has to be noted that two important aspects are different in comparison to the present study. First, Harrington et al. assessed the retest reliability by the overlap of activated voxels between the first and second run. This approach uses a less stringent criterion than our approach since it does not require similar brain activation in both runs, but only consistently activation above arbitrary chosen significance thresholds. Second, they compared brain activity differences in the scenes paradigm not between “new” and “old” items, but between scenes and noise images, that is, they used a low-level baseline. This leads to higher *t*-values that are typically associated with higher reliability values. These differences might explain why Harrington and colleagues report high reliability values also for MTL activity.

A number of previous studies investigated which factors influence retest reliability. Retest reliability is influenced by numerous factors such as task design, statistical contrast, thresholding, scanner noise, coregistration error, and subject motion (30, 37). Furthermore, reproducibility of individual subject activation maps is often highly variable, indicating that reliable results might be obtained only in some subjects. These individual differences are associated with individual differences in the global temporal signal-to-noise ratio (38). The chosen task itself however has been shown to be one of the most important contributor to single subject reliability, having more influence than many other factors (30). Therefore our results might be interpreted that the novelty encoding paradigms we tested in the present study, although they seem to be commonly applied even in the clinical context, have limited reliability in typical fMRI measurements, at least for the words and fractals versions. One might further improve the reliability of these tasks by changing technical aspects of the measurements, for instance by applying more appropriate MR imaging sequences (39). However, instead of primarily changing methodological aspects of the design, it might be more promising to use different task implementations, for instance by additionally introducing low-level baseline conditions (e.g., scrambled noise images) so that activation and baseline condition differ more strongly in their activation level. Also the introduction of different memory tasks, e.g., explicit memory encoding tasks or recognition task, might improve the overall retest reliability. Future projects will have to investigate these aspects in more detail, in particular with respect to the properties and informative value of different reliability metrics. Overall, the imaging community has to further develop comprehensive guides for the development of robust test-retest paradigms.

## Conflict of Interest Statement

The authors declare that the research was conducted in the absence of any commercial or financial relationships that could be construed as a potential conflict of interest.

## Figures and Tables

**Figure A1:**
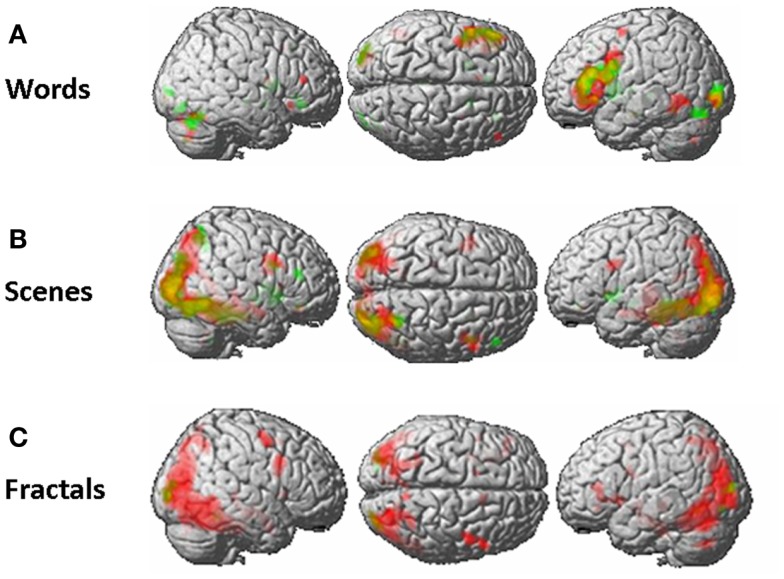
**Overlap of activated brain regions of the first (red) and second (green) measurement, as assessed by a mixed-effects group analysis (contrast: “new > old”), for the words paradigm (A), the scenes paradigm (B), and the fractals paradigm (C)**. T-maps are thresholded at a rather liberal significance level (*p* < 0.01, uncorrected) to better illustrate the overlap of activated brain networks.

## Appendix

### Creation of stimulus material

#### Words

We used German nouns and verbs with medium word frequency in the German language as indicated in the Celex Word Database of the Max Planck Institute for Linguistics in Nijmwegen (http://www.ru.nl/celex). The words had two syllables and were four to seven letters long. Half of the words were verbs, the other half nouns. Words were presented in black capital letters on gray background. Image size was 354 × 354 pixels. The participants were instructed to indicate whether a presented word was a noun or a verb.

#### Scenes

The second group of stimuli consisted of photographs of indoor and outdoor scenes. The images used were collected from private and internet sources (e.g., http://www.hintergrundbilder-pc.de, http://www.flickr.com/.) All photographs were resized to 354 × 354 pixels. Half of the photographs depicted indoor scenes, the other half outdoor scenes. The task assigned to this stimulus class was to indicate whether the pictures showed indoor or outdoor scenes.

#### Fractals

The fractals were created using Apophysis 2.02 for Linux (http://apophysis.org/index.html). All pictures were scaled to 354 × 354 pixels and 50% were converted to black and white using Irfanview 4.25 for Microsoft Windows^®^, Copyright by Irfan Skiljan (http://www.irfanview.de/). The task assigned to this stimulus class was to differentiate between colored and black and white pictures.

## References

[1] Beaumont JG Future research directions in laterality. Neuropsychol Rev (1997) 7:107–26 10.1023/B:NERV.0000005947.20270.80 9339455

[2] Crosson B McGregor K Gopinath KS Conway TW Benjamin M Chang Y-L Functional MRI of language in aphasia: a review of the literature and the methodological challenges. Neuropsychol Rev (2007) 17:157–77 10.1007/s11065-007-9024-z 17525865 PMC2659355

[3] Knecht S Flöel A Dräger B Breitenstein C Sommer J Henningsen H Degree of language lateralization determines susceptibility to unilateral brain lesions. Nat Neurosci (2002) 5(7):695–9 12055632 10.1038/nn868

[4] Sommer IEC Ramsey NF Kahn RS Language lateralization in schizophrenia, an fMRI study. Schizophr Res (2001) 52:57–67 10.1016/S0920-9964(00)00180-8 11595392

[5] Seghier ML Josse G Leff AP Price CJ Lateralization is predicted by reduced coupling from the left to right prefrontal cortex during semantic decisions on written words. Cereb Cortex (2011) 21:1519–31 10.1093/cercor/bhq203 21109578 PMC3116735

[6] Stephan KE Marshall JC Friston KJ Rowe JB Ritzl A Zilles K Lateralized cognitive processes and lateralized task control in the human brain. Science (2003) 301:384–6 10.1126/science.1086025 12869765

[7] Jansen A Flöel A Menke R Kanowski M Knecht S Dominance for language and spatial processing: limited capacity of a single hemisphere. Neuroreport (2005) 16:1017–21 10.1097/00001756-200506210-00027 15931080

[8] Jansen A Deppe M Schwindt W Mohammadi S Sehlmeyer C Knecht S Interhemispheric dissociation of language regions in a healthy subject. Arch Neurol (2006) 63:1344–6 10.1001/archneur.63.9.1344 16966524

[9] Jansen A Müller S Bedenbender J Krach S Paulus FM Kircher T Determination of crossed language dominance: dissociation of language lateralization within the temporoparietal cortex. Neurocase (2013) 19:348–50 10.1080/13554794.2012.667129 22512289

[10] Bennett CM Miller MB How reliable are the results from functional magnetic resonance imaging? Ann N Y Acad Sci (2010) 1191:133–55 10.1111/j.1749-6632.2010.05446.x 20392279

[11] Atri A O’Brien JL Sreenivasan A Rastegar S Salisbury S DeLuca AN Test-retest reliability of memory task functional magnetic resonance imaging in Alzheimer disease clinical trials. Arch Neurol (2011) 68:599–606 10.1001/archneurol.2011.94 21555634 PMC3291175

[12] Putcha D O’Keefe K LaViolette P O’Brien J Greve D Rentz DM Reliability of functional magnetic resonance imaging associative encoding memory paradigms in non-demented elderly adults. Hum Brain Mapp (2011) 32:2027–44 10.1002/hbm.21166 21259385 PMC3551453

[13] Harrington GS Tomaszewski Farias S Buonocore MH Yonelinas AP The intersubject and intrasubject reproducibility of FMRI activation during three encoding tasks: implications for clinical applications. Neuroradiology (2006) 48:495–505 10.1007/s00234-006-0083-2 16703360

[14] Golby AJ Poldrack RA Brewer JB Spencer D Desmond JE Aron AP Material-specific lateralization in the medial temporal lobe and prefrontal cortex during memory encoding. Brain (2001) 124:1841–54 10.1093/brain/124.9.1841 11522586

[15] Jansen A Sehlmeyer C Pfleiderer B Sommer J Konrad C Zwitserlood P Assessment of verbal memory by fMRI: lateralization and functional neuroanatomy. Clin Neurol Neurosurg (2009) 111:57–62 10.1016/j.clineuro.2008.08.005 18922628

[16] Powell HW Koepp MJ Richardson MP Symms MR Thompson PJ Duncan JS The application of functional MRI of memory in temporal lobe epilepsy: a clinical review. Epilepsia (2004) 45:855–63 10.1111/j.0013-9580.2004.41603.x 15230713

[17] Gabrieli JDE Cognitive neuroscience of human memory. Annu Rev Psychol (1998) 49:87–115 9496622 10.1146/annurev.psych.49.1.87

[18] Hwang DY Golby AJ The brain basis for episodic memory: insights from functional MRI, intracranial EEG, and patients with epilepsy. Epilepsy Behav (2006) 8:115–26 10.1016/j.yebeh.2005.09.009 16278097

[19] Poldrack RA Gabrieli JD Memory and the brain: what’s right and what’s left? Cell (1998) 93:1091–3 9657140 10.1016/s0092-8674(00)81451-8

[20] Oldfield RC The assessment and analysis of handedness: the Edinburgh inventory. Neuropsychologia (1971) 9:97–113 10.1016/0028-3932(71)90067-4 5146491

[21] Maldjian JA Laurienti PJ Kraft RA Burdette JH An automated method for neuroanatomic and cytoarchitectonic atlas-based interrogation of fMRI data sets. Neuroimage (2003) 19:1233–9 10.1016/S1053-8119(03)00169-1 12880848

[22] Eickhoff SB Stephan KE Mohlberg H Grefkes C Fink GR Amunts K A new SPM toolbox for combining probabilistic cytoarchitectonic maps and functional imaging data. Neuroimage (2005) 25:1325–35 10.1016/j.neuroimage.2004.12.034 15850749

[23] Golby AJ Poldrack RA Illes J Chen D Desmond JE Gabrieli JDE Memory lateralization in medial temporal lobe epilepsy assessed by functional MRI. Epilepsia (2002) 43:855–63 10.1046/j.1528-1157.2002.20501.x 12181004

[24] Jansen A Menke R Sommer J Förster AF Bruchmann S Hempleman J The assessment of hemispheric lateralization in functional MRI – robustness and reproducibility. Neuroimage (2006) 33:214–7 10.1016/j.neuroimage.2006.06.019 16904913

[25] Shrout PE Fleiss JL Intraclass correlations: uses in assessing rater reliability. Psychol Bull (1979) 86:420–8 18839484 10.1037//0033-2909.86.2.420

[26] Caceres A Hall DL Zelaya FO Williams SC Mehta MA Measuring fMRI reliability with the intra-class correlation coefficient. Neuroimage (2009) 45:758–68 10.1016/j.neuroimage.2008.12.035 19166942

[27] Fliessbach K Rohe T Linder NS Trautner P Elger CE Weber B Retest reliability of reward-related BOLD signals. Neuroimage (2010) 50:1168–76 10.1016/j.neuroimage.2010.01.036 20083206

[28] Weber B Fliessbach K Lange N Kugler F Elger CE Material-specific memory processing is related to language dominance. Neuroimage (2007) 37:611–7 10.1016/j.neuroimage.2007.05.022 17574870

[29] Seghier ML Kherif F Josse G Price CJ Regional and hemispheric determinants of language laterality: implications for preoperative fMRI. Hum Brain Mapp (2011) 32:1602–14 10.1002/hbm.21130 20814960 PMC3193373

[30] Gorgolewski KJ Storkey AJ Bastin ME Whittle I Pernet C Single subject fMRI test-retest reliability metrics and confounding factors. Neuroimage (2013) 69:231–43 10.1016/j.neuroimage.2012.10.085 23153967

[31] Avila C Barrós-Loscertales A Forn C Mallo R Parcet M-A Belloch V Memory lateralization with 2 functional MR imaging tasks in patients with lesions in the temporal lobe. AJNR Am J Neuroradiol (2006) 27:498–503 16551984 PMC7976998

[32] Deblaere K Backes WH Tieleman A Vandemaele P Defreyne L Vonck K Lateralized anterior mesiotemporal lobe activation: semirandom functional MR imaging encoding paradigm in patients with temporal lobe epilepsy – initial experience. Radiology (2005) 236:996–1003 10.1148/radiol.2363040780 16118173

[33] Paulus FM Krach S Albrecht AG Jansen A Potential bias in meta-analyses of effect sizes in imaging genetics. Schizophr Bull (2013) 39(3):501–3 10.1093/schbul/sbt035 23474966 PMC3627758

[34] Paulus FM Bedenbender J Krach S Pyka M Krug A Sommer J Association of rs1006737 in CACNA1C with alterations in prefrontal activation and fronto-hippocampal connectivity. Hum Brain Mapp (2013). 10.1002/hbm.22244 23404764 PMC6869796

[35] Paulus FM Krach S Bedenbender J Pyka M Sommer J Krug A Partial support for ZNF804A genotype-dependent alterations in prefrontal connectivity. Hum Brain Mapp (2013) 34(2):304–13 10.1002/hbm.21434 22042765 PMC6870005

[36] Jansen A Krach S Krug A Markov V Thimm M Paulus FM The effect of G72 genotype on neural correlates of memory encoding and retrieval. Neuroimage (2010) 53(3):1001–6 10.1016/j.neuroimage.2009.12.018 20005295

[37] Bennett CM Miller MB fMRI reliability: influences of task and experimental design. Cogn Affect Behav Neurosci (2013). 10.3758/s13415-013-0195-1 23934630

[38] Raemaekers M Vink M Zandbelt B van Wezel RJA Kahn RS Ramsey NF Test-retest reliability of fMRI activation during prosaccades and antisaccades. Neuroimage (2007) 36:532–42 10.1016/j.neuroimage.2007.03.061 17499525

[39] Olman CA Davachi L Inati S Distortion and signal loss in medial temporal lobe. PLoS ONE (2009) 4(12):e8160 10.1371/journal.pone.0008160 19997633 PMC2780716

